# Case III—Anemia and Anasarca

**Published:** 1871-03

**Authors:** 


Case 3—Anemia and Anasarca.—Admitted Sunday, the 12th inst., presenting the following conditions: A bloodless hue of the surface; lips pale, and considerable degree of œdema in the extremities, and general weakness. From the fact that this condition had remained for several weeks without improvement, and the secretion of urine being diminished, albuminuria was suggested. The urine was tested by heat and nitric acid, but no trace of albumen was developed, which would indicate that there was no granular disease of the kidneys.

The patient also complained of a tightness across the chest, and some cough, but was not examined for pulmonary or cardiac disease at the time.

On auscultation we find in each full inspiration coarse, rough, bronchial sounds; over the cardiac region we can distinguish no valvular roughness or murmur, but the sounds are short, quick, and distant. The systole is shorter than natural, yet distinct and clearly perceived; apex impulse absent.

On percussion, no marked dulness on either side till we reach the cardiac space on the left side. Here dulness begins pretty high up and extends to the bottom of the chest, and over the region of the spleen there is nearly double the normal extent of dulness. The intercostal spaces are well filled out and both hypochondriac regions full. There is probably some pericardial effusion, but we may attribute most of the enlargement on this side to the spleen. On the right side, also, there is dulness over a little greater than the normal vertical depth, showing that there is probably moderate enlargement of the liver.

A combination of causes has apparently contributed to the development of a degree of fatty degeneration in the liver, and spleen, and there may also be, to some extent, the same change in the structure of the heart, a slow change in the nutrition of these organs diminishing their ability to contribute their proper influence in the process of assimilation, which would account for his gradually assuming this anemic condition.

There is not that degree of enlargement in the parts referred to which would produce effusion from obstruction to the portal circulation, in which case abdominal dropsy would precede general œdema.

The bronchial sounds are a part of an old, simple bronchial irritation which many persons are subject to during the cold season, but which involves no structural change, and is a minor matter.

On admission, observing the quick, agitated movements of the heart, oppression in the chest, and scanty urine, he was directed to have a combination of digitalis and scutellaria, as follows: Fl. ext. scutellaria ℥iij, tinct. digitalis ℥i.—M. One teaspoonful of this to be given four times a-day, to increase the amount of urine, and give steadiness and force to the heart's action. I would advise a continuation of the same, but for a further influence on the assimilative function, he should have, in addition, some tonic and alterative. A good addition would be bismuth 6 grs., lupuline 2 grs., sub-carb. of iron 4 grs.—M., to be taken half an hour after each meal. He should have a simple, easily assimilated diet. This case, in many of its features, resembles those described as leucocythæmia.

